# Effects of a Phytogenic Mycotoxin Detoxifier on Oxidative Status, Health, and Performance in Dairy Sheep

**DOI:** 10.3390/toxins17080425

**Published:** 2025-08-21

**Authors:** Georgios I. Papakonstantinou, Christos Eliopoulos, Eleftherios Meletis, Insaf Riahi, Evangelos-Georgios Stampinas, Dimitrios Arapoglou, Dimitrios Gougoulis, Konstantina Dimoveli, Dimitrios Filippou, Alexandros Manouras, Nikolaos Tsekouras, Lampros Fotos, Polychronis Kostoulas, Georgios Christodoulopoulos, Vasileios G. Papatsiros

**Affiliations:** 1 Clinic of Medicine, Faculty of Veterinary Medicine, University of Thessaly, 43100 Karditsa, Greece; dgoug@uth.gr (D.G.); kdimoveli@uth.gr (K.D.); nitsekou@vet.uth.gr (N.T.); lampros.fotos@terracert.gr (L.F.); vpapatsiros@vet.uth.gr (V.G.P.); 2 Institute of Technology of Agricultural Products, Hellenic Agricultural Organization-Demeter (HAO-Demeter), 14123 Athens, Greece; chris_eliopoulos@hotmail.com (C.E.); dimarap@yahoo.com (D.A.); 3 Faculty of Public and One Health, University of Thessaly, 43100 Karditsa, Greece; elmeletis@uth.gr (E.M.); pkost@uth.gr (P.K.); 4 BIŌNTE Animal Nutrition, 43204 Reus, Spain; insaf.riahi@bionte.com; 5 Laboratory of Anatomy, Histology & Embryology, Veterinary School, University of Thessaly, 43100 Karditsa, Greece; estampinas@vet.uth.gr; 6 Agricultural Cooperative of Cattle Breeders and Sheep Breeders Western Thessaly, 43060 Thessaly, Greece; dimfilippou1993@gmail.com (D.F.); almanouras@hotmail.com (A.M.); 7 Department of Animal Science, Agricultural University of Athens, 11855 Athens, Greece; gc@aua.gr

**Keywords:** sheep, mycotoxin, mastitis, oxidative, liver, detoxifier, curcumin, silymarin

## Abstract

Mycotoxins are common feed contaminants that can affect the health, immune function, and productivity of ruminants by causing oxidative stress and organ dysfunction. In this field study, the effects of a phytogenic multicomponent mycotoxin detoxifier on oxidative status, liver function, udder health, and productive parameters were investigated in dairy ewes. One hundred clinically healthy ewes were randomly assigned to either a control group or a treatment group, with the latter receiving 1.5 kg/ton of the detoxifier over a 90-day period during lactation. The detoxifying agent contained adsorptive clays as well as phytogenic ingredients such as silymarin and curcumin, which are known for their hepatoprotective and antioxidant properties. Blood, milk, and colostrum samples were collected and analyzed for oxidative stress markers (TBARS and protein carbonyl (CARBS)), total antioxidant capacity (TAC), liver enzymes (ALT, AST, and ALP), and milk quality parameters (fat, protein, and solid content). Clinical assessments included mastitis scoring, udder inflammation, and fecal consistency. The treated ewes showed a statistically significant reduction in blood plasma and milk oxidative stress markers and liver enzyme levels while at the same time improving the fat and solid content of the milk. The incidence and severity of mastitis, udder reddening, and lactation abnormalities were lower in the treatment group. Brix refractometry indicated improved colostrum quality in the treated ewes. These results suggest that the detoxifier improved the oxidative balance, liver function, and overall health and productivity of dairy ewes under field conditions, supporting its use as a practical nutritional measure.

## 1. Introduction

Mycotoxins are secondary metabolites generated by fungi (molds) and are commonly found in food and animal feed across the globe. The most significant plant pathogens responsible for contaminating food crops and producing mycotoxins in feed are species of *Aspergillus* and *Fusarium*. Even at low concentrations, mycotoxins pose serious health risks to both humans and animals. The most common mycotoxins in animal feed are aflatoxins (Afs; aflatoxin B1-AF-B1 and aflatoxin B2-AF-B2); ochratoxin A (OTA); trichothecenes, such as deoxynivalenol (DON), T-2 toxin (T-2), and HT-2 toxin (HT-2); fumonisins (FUBs; fumonisin B1-FUB1 and fumonisin B2 FUB-2); and zearalenone (ZEN). Mycotoxins are a critical risk factor for sheep health as they contaminate cereal grains, which are a common component of sheep feed, on a large scale. Consuming mycotoxins, even in minimal amounts, can result in acute, subacute, or chronic illnesses—collectively known as mycotoxicosis—which contribute to substantial economic losses. Mycotoxin exposure through food can seriously impair the immune, reproductive, and respiratory systems and damage vital organs such as the intestines, kidneys, and liver. In sheep specifically, mycotoxins can lead to (a) decreased feed intake, (b) gastrointestinal (GI) dysfunction and oxidative stress, (c) various reproductive and neonatal disorders, and (d) health complications in offsprings [1].

The primary strategy for preventing and controlling mycotoxins involves the use of detoxifying agents added to animal feed. These commercially available feed additives are typically classified into two main types: (a) adsorbents (mycotoxin binders) and (b) biotransformation agents (mycotoxin modifiers). Both types aim to reduce the absorption of mycotoxins in the GI tract and prevent their entry into the bloodstream [2,3]. Adsorbents bind mycotoxins in the GI tract, promote their excretion via feces, and thus prevent their absorption. In contrast, biotransforming agents convert mycotoxins into less harmful or non-toxic metabolites with the help of microorganisms or enzymes [4]. The transformed mycotoxins are subsequently excreted from the body, leading to decreased accumulation in target organs. Mycotoxin binders and modifiers frequently include supplementary ingredients such as clays, yeast derivatives, phytogenic compounds, and herbal extracts, which help enhance nutrient absorption, strengthen the immune system, support intestinal health, and mitigate oxidative stress [4]. Some also contain beneficial nutrients such as fiber or nucleotides to further support detoxification [5]. Mycotoxin binders or modifiers typically consist of multiple ingredients—such as clay, yeast derivatives, phytogenics, and herbs—designed to enhance absorption, support immune function, promote gut health, and reduce oxidative stress. Some also include essential components like fiber and nucleotides to boost the body’s detoxification capacity.

The detoxifying agent used in this study combines several functional components, including adsorptive clays (bentonite and sepiolite), yeast derivatives, and two phytogenic compounds (silymarin and curcumin), each with different mechanisms of action. Bentonite and sepiolite are adsorptive clays with a large surface area and cation exchange capacity, allowing them to bind polar mycotoxins—especially aflatoxins—in the gastrointestinal tract and limiting their systemic absorption and toxicity [4]. Silymarin is a natural extract derived from *Silybum marianum* (commonly known as milk thistle) and is composed of four primary isomeric constituents: silybin, isosilybin, silydianin, and silychristin [6]. In farm animals, silymarin has been reported to improve performance parameters and the quality of animal-source foods, support liver function throughout the production cycle, improve intestinal health and morphology, and reduce the incidence of intestinal pathogens [7]. Moreover, multiple studies have recognized silymarin as an effective means of alleviating oxidative stress during the early lactation period in ewes, primarily due to its antioxidant and hepatoprotective properties [8,9,10,11,12,13]. Curcumin, a polyphenol derived from *Curcuma longa*, is widely known for its use in traditional Asian medicine [14]. When included in the diet of dairy sheep, curcumin shows strong anti-inflammatory and antioxidant effects. It has been associated with increased milk production, increased total antioxidant content, reduced protein oxidation in milk, and reduced somatic cell counts [15]. In addition, curcumin supplementation has shown positive effects in sheep suffering from subclinical mastitis [16]. Yeast derivatives, including yeast cell walls and hydrolyzed yeast, fulfill a dual function by binding various mycotoxins (e.g., zearalenone and aflatoxins) and modulating the immune response through β-glucans and mannans, which support gut integrity and microbial balance [4,5]. The inclusion of these components supports a multi-faceted approach to mycotoxin control by reducing toxin bioavailability, protecting liver function, and increasing the animal’s antioxidant capacity.

Mycotoxins are commonly found in sheep across the globe and can cause acute, subacute, or chronic forms of mycotoxicosis, resulting in considerable economic losses. This study aims to evaluate the impact of a multi-component mycotoxin detoxifier on the redox balance, health status, and performance of dairy sheep under field conditions.

## 2. Results

### 2.1. Clinical Performance Parameters

No adverse effects related to the administration of the detoxifier were observed during the trial. All treated animals maintained good general health and appetite, with no signs of intolerance or toxicity recorded throughout the study period. The assessment of clinical parameters between trial groups is shown in Table 1. Statistically significant differences were observed between the control and experimental groups. Notable decreases occurred in redness, consistency, and formation/regression scores, indicating a positive effect of the detoxifier on udder health and lactation status. Table S1 in the supplementary material shows the detailed evaluation of clinical symptoms, lameness, mastitis, and milk composition in the ewes by treatment group.

### 2.2. Colostrum/Milk Quality Parameters

The laboratory exams that were carried out in the colostrum and milk showed that group T2 (treated ewes) had significantly higher fat and total solids values and a greater proportion of ‘good’-quality colostrum based on Brix, as presented in Table 2. Based on Brix refractometry, the proportion of animals with “Good” quality (>26%) was 64% (32/50) in the treated group (T2) compared to 24% (12/50) in the control group (T1). Conversely, 30% (15/50) of the T1 animals had “Poor” colostrum quality (<22%) compared to only 6% (3/50) in the T2 group, indicating a significant improvement in passive immunotransfer potential in the treated ewes.

### 2.3. Quantification of Mycotoxins in Feed

The concentration of aflatoxin B1 (AF-B1) in feed samples was measured at 26.66 μg/kg, which is above the maximum level of 20 μg/kg set by the European Commission for dairy cattle feed [17]. Other mycotoxins such as FUM (B1 + B2), OTA, DON, ZEN, and T2/HT-2 were detected in quantities that were well below the respective safety thresholds. The laboratory exams for mycotoxins in feed samples showed the detection of AF-B1, as presented in Table 3.

### 2.4. Blood Laboratory Exams for Redox and Liver Biomarkers

Both groups were randomized before the study and matched on the basis of health status and body weight. All animals were exposed to the same naturally contaminated feed containing AFB1. The control group (T1) received no treatment, while the treatment group (T2) received the detoxifying agent. These conditions ensured comparable initial exposure and health status so that the differences observed at the end of the experiment could be attributed to the detoxifying agent. Table 4 summarizes the plasma levels of thiobarbituric-acid-reactive substances (TBARS, μM MDA), protein carbonyls (CARB, nmol/mg protein), and total antioxidant capacity (TAC, mM Trolox Equivalent) in the control and experimental groups. The levels of TBARS were significantly reduced (*p* < 0.05) in the experimental group compared to the control group, decreasing from 3.84 to 2.17 μM MDA. Similarly, CARB content showed a significant reduction (*p* < 0.05) of 32.43% between the two groups. In contrast, TAC levels exhibited a modest increase, rising from 0.79 to 0.87 mM Trolox Equivalent. However, this difference was not statistically significant (*p* > 0.05).

Table 5 presents the results that concern sheep examined for biochemical parameters of ALT, AST, and ALP. Specifically, the ALT content recorded a significant reduction (*p* < 0.05) between pre-feeding and post-feeding periods, with the corresponding values ranging from 38.14 to 17.73 U/L. AST levels were found to be statistically significant (*p* < 0.05) decreased among the examined periods by 27.92%. Finally, ALP presented a similar behavior since the latter’s presence revealed a statistically significant (*p* < 0.05) reduction of 28.53% at the end of the feeding trial.

According to Table 6, the levels of TBARS and CARBS were gradually reduced in milk samples during the trial. Specifically, TBARS recorded a statistically significant (*p* < 0.05) reduction of 26.43% between the pre-feeding and during-feeding periods. Furthermore, TBARS’ presence was found to be significantly (*p* < 0.05) reduced at the end of the trial compared with the pre-feeding period, with the corresponding values ranging from 21.03 to 9.88 μM MDA. CARBS presented a similar pattern, since their content was statistically significant (*p* < 0.05) reduced among all the examined periods. CARBS’ levels were significantly (*p* < 0.05) depleted by 31.25% during the feeding period, whereas the post feeding period recorded a significant (*p* < 0.05) reduction of 50% compared to the beginning of the experiment.

## 3. Discussion

Exposure of ruminants to complex mycotoxin mixtures is increasingly recognized as a significant risk to animal health and productivity, even in species with a natural detoxification capacity [21,22]. This in vivo study demonstrated that supplementation with a phytogenic mycotoxin detoxifier significantly improved oxidative status, liver enzyme profiles, udder health, and the milk composition in lactating dairy ewes. During the trial, the feed analysis revealed that the concentration of aflatoxin B1 (AF-B1) reached 26.66 μg/kg, exceeding the maximum permitted level of 20 μg/kg for dairy cattle set by EU regulations [17]. This finding confirms that the animals were exposed to mycotoxin contamination, which is considered unsafe, and emphasizes the practical relevance of this study. The detoxifying agent used in this study contains several active components, each selected for their specific biological role in mitigating the harmful effects of mycotoxins. Bentonite and sepiolite are adsorptive clays with high cation exchange capacity, allowing them to bind polar mycotoxins such as aflatoxins in the gastrointestinal tract and limit their systemic absorption [4]. Yeast derivatives, including hydrolyzed yeast and yeast cell wall fragments, contribute to the binding of mycotoxins, especially zearalenone and aflatoxins, while supporting gut integrity and modulating immune responses through β-glucans and mannans [4,5]. The phytogenic compounds silymarin and curcumin act primarily through antioxidant and hepatoprotective mechanisms. Silymarin stabilizes the hepatocyte membranes, reduces lipid peroxidation, and strengthens the antioxidant enzymes in the liver, thereby protecting the liver from toxin-induced oxidative damage [6,7,8,9,10,11,12,13]. Curcumin has anti-inflammatory and antioxidant effects, increases the expression of antioxidant enzymes, and reduces oxidative stress markers in milk and plasma [15,16]. Together, these components provide synergistic and multi-layered protection: they absorb toxins in the gut, protect and improve liver function, reduce systemic oxidative stress, and maintain immunocompetence. This combination mechanism is consistent with previous studies demonstrating improvements in animal health, productivity, and milk quality when such ingredients are used as part of an anti-mycotoxin strategy in ruminants [23,24,25].The clinical, biochemical, and oxidative stress improvements observed in the treated group are therefore particularly meaningful as they occurred under documented aflatoxin exposure. Our results are not only consistent with previous research in cattle [23,24] and goats [25] but also provide new insights into the use of phytogenic detoxifiers in dairy sheep under commercial farm conditions. To our knowledge, this is the first field study in dairy sheep to show an improvement in the udder health, milk solids, and IgG quality of colostrum with a combined phytogenic and adsorbent detoxifier.

Numerous studies have documented the detrimental effects of mycotoxins on ruminant health, particularly their ability to disrupt gastrointestinal and reproductive functions [26,27]. Such disruption often leads to reduced feed intake, impaired milk quality and yield, reduced fertility, and increased incidence of lameness, mastitis, and milk contamination [22,28,29,30,31,32,33]. It is also known that mycotoxins weaken the immune defense and make animals more susceptible to prolonged and severe infections, such as mastitis [32,33,34]. Mastitis is a significant health issue affecting productive ewes globally, characterized by inflammation of the mammary glands. Mastitis is a significant health issue in productive ruminants. A recent peer-reviewed study found that feed contaminated with mycotoxins was significantly associated with increased rates of clinical and subclinical mastitis in dairy cows, accompanied by alterations in mineral and antioxidant status [35]. These toxins impair immune function via several mechanisms, including reduced phagocytic activity, impaired cell-mediated and humoral immunity, and impaired gut integrity [36,37,38]. In our study, the presence of aflatoxin B1 in the feed indicated a significant risk of mycotoxicosis. It is noteworthy that the treated animals showed a significant improvement in clinical health, as evidenced by a lower incidence of disease symptoms and a reduction in udder-related clinical parameters. By mitigating these effects, the phytogenic detoxifier appears to improve both physiological function and production performance. The reduced incidence of mastitis and associated inflammation is consistent with reports of mycotoxin-induced immunosuppression and the known anti-inflammatory effects of curcumin [15,16] and silymarin [8,11]. Improved mammary gland integrity, as reflected in milk composition and somatic cell trends, could contribute to improved milk quality and reduced disease incidence.

Both blood plasma and milk serum samples showed significant improvements in oxidative stress biomarkers after treatment. The levels of TBARS and CARBS were significantly reduced in the treated group, indicating less lipid and protein oxidation. These results suggest that the detoxifying agent strengthens antioxidant defenses and mitigates oxidative damage at both the systemic and infant levels. The improved redox balance is likely due to the presence of curcumin and silymarin, compounds known for their free radical scavenging and anti-inflammatory properties. Bozakova and Ivanov [39] report that silymarin contributes to improved sheep welfare through its antioxidant and detoxifying effects and promotes growth and milk production. Similarly, Jaguezeski et al. [40] found that curcumin supplementation improved milk quality, reduced lipid peroxidation in milk, and enhanced both anti-inflammatory and antioxidant responses in dairy sheep. In a separate study, Jaguezeski et al. [15] also showed that curcumin supplementation increased milk production and the content of unsaturated fatty acids, especially oleic acid, in sheep’s milk. Overall, the results of the present study are consistent with previous research, emphasizing the positive effects of silymarin and curcumin on improving oxidative balance, animal welfare, and milk quality in sheep.

Various mycotoxins have been reported to have negative effects on milk composition and overall milk quality. These changes are often associated with immune suppression, GI damage, or irregular feed intake—all factors that can reduce the availability of precursors needed to synthesize important milk components [41,42]. Mycotoxins such as DON and fumonisins have been associated with altered characteristics in cheese production, including reduced curd firmness and delayed curd ripening time. In addition, feed contaminated with several mycotoxins has also been associated with reduced curd quality and consistency [42]. Our results are consistent with these previous studies, as one of the most notable results of our trial was the significant improvement in milk composition observed in the T2 group. Fat content and solids yield (SY) were significantly increased, suggesting improved nutrient intake and metabolic function in the treated sheep. These effects are likely due to the active ingredients of the detoxifier—silymarin and curcumin—in supporting liver and antioxidant activity [39]. Such improvements are important for both animal performance and product value. A higher fat content contributes to better energy availability during lactation, which is crucial for maintaining milk yield and avoiding a negative energy balance. In dairy farming, a higher solid content is also associated with better cheese yield and curd consistency, which increases the economic value of milk. Similarly, the higher proportion of “Good”-quality colostrum in the T2 group indicates improved IgG content, which supports neonatal immunity and early lamb survival—an important productivity factor in commercial sheep farms.

Brix refractometry was used to measure colostrum quality. It has been reported to be highly correlated to direct measures of IgG by ELISA in sheep colostrum samples [43]. Colostrum quality was significantly better in the treated group (T2). A greater proportion of samples exceeded the 26% Brix threshold, indicating improved transfer of IgG to newborn lambs—a key determinant of early-life immunity. This improvement could be related to a better general health status and lower inflammatory stress in the ewes. Furthermore, the phytogenic compounds included in the detoxifier may enhance these immunomodulatory effects, as they have been effectively used to promote sheep welfare through their antioxidant, hepatoprotective, anti-stress, and detoxifying properties, as well as their ability to stimulate growth and milk production [39].

In our study, the control group (T1) had ALT values slightly above or close to the upper normal values (~33 and ~126 U/L, respectively) for healthy sheep. The ALP values were within the normal range. The reductions in ALT and AST levels observed after treatment in the T2 group brought the animals into clearly normal physiological ranges [44]. These changes are not only statistically significant but also clinically meaningful and indicate the elimination of the subclinical liver stress probably caused by chronic AFB1 exposure. This finding is particularly important as the liver plays a central role in the metabolism and degradation of mycotoxins. Most mycotoxins are biotransformed in the liver, a process that can generate reactive oxygen species (ROS) and cause oxidative damage to liver tissue. Elevated liver enzymes in the blood usually indicate cell damage and loss of membrane integrity of hepatocytes [44]. The observed reductions in ALT, AST, and ALP therefore indicate not only less damage to the liver cells but also improved efficiency and integrity of the liver in detoxification processes.

Maintaining liver function is critical in ruminants exposed to dietary mycotoxins, as impaired liver capacity can lead to systemic accumulation of toxins and secondary damage to the immune, reproductive, and gastrointestinal systems [45]. The phytogenic components of the detoxifier—particularly silymarin and curcumin—have been shown in previous studies to stabilize hepatocyte membranes, increase antioxidant enzyme activity, and reduce lipid peroxidation, thereby protecting liver tissue from mycotoxin-induced damage [39,40]. This hepatoprotective effect likely contributed to the broader metabolic improvements observed in the treated animals. By supporting liver resilience, the detoxifying agent may have enabled more effective nutrient metabolism and energy balance during the demanding lactation period. These benefits are particularly important under field conditions, where low-level, chronic mycotoxin exposure is common and animals are often metabolically stressed.

In addition, the reduction in liver stress correlates with the systemically observed improvement in oxidative status, as oxidative stress is closely associated with liver dysfunction. According to Celi [46] and Celi & Gabai [47], the liver is a major source and target of oxidative damage in ruminants, especially during dietary or environmental challenges. By simultaneously lowering oxidative markers (such as TBARS and CARBS) and liver enzymes, the detoxifier has a dual effect: it reduces toxin exposure and supports systemic redox balance. Such physiological improvements can translate into tangible productivity benefits. For example, Gatellier et al. [45] and Di Trana et al. [44] reported that animals with a higher antioxidant capacity induced by either phytogenic supplementation or diet had better product quality—be it meat or milk—emphasizing the role of oxidative status in determining productive efficiency and product value.

The integration of clinical health improvements, restored liver function, improved oxidative balance, and better milk quality suggests that this phytogenic detoxification agent offers a robust, multi-center strategy for the treatment of mycotoxicosis in dairy sheep. These effects are particularly valuable under practical farm conditions where animals are routinely exposed to fluctuating feed quality and co-occurring mycotoxins. In addition to its binding activity, the detoxifier acts as a functional feed additive, providing antioxidant, immunomodulatory, and metabolic support to enhance animal health and resilience on multiple physiological axes. A previous study using the same anti-mycotoxin agent in dairy cows demonstrated its effectiveness against mycotoxins through a triple-action mechanism [23]. The product, which integrates adsorptive materials, phytogenic extracts, and post-biotic compounds, exhibited a broad mode of action with high binding efficiency for multiple mycotoxins, including AFB1, T-2, and HT-2 toxins. This mechanism involves not only adsorption but also the reduction in mycotoxin effects through hepatoprotective, antioxidant, and anti-inflammatory activities—which are primarily attributed to the inclusion of phytogenic extracts in the formulation [23].

## 4. Conclusions

The present study provides strong evidence that the phytogenic mycotoxin detoxifier used can improve oxidative balance, liver function, udder health, and milk quality in dairy sheep exposed to natural mycotoxin contamination. The integration of adsorbing minerals, silymarin, curcumin, and yeast-derived compounds may mitigate the subclinical effects of mycotoxins under field conditions. However, the limitations of this study, including the relatively short duration and reliance on naturally occurring mycotoxin levels, should be considered when interpreting the results. Therefore, future research should focus on longer-term trials with different flocks, controlled mycotoxin exposure models, and dose–response studies to better characterize the individual contributions of each detoxification component. In addition, the effects of such interventions on newborn lamb health, immune system development, and long-term productivity could be of great importance. Overall, phytogenic detoxifiers show promising results as feed additives in production systems for ruminants struggling with persistent mycotoxin problems.

## 5. Materials and Methods

### 5.1. Compliance with Ethical Standards

All procedures related to animal care and handling were reviewed and approved by the Institutional Ethical Committee of the University of Thessaly (approval No. 102, dated 16 November 2021).

### 5.2. Trial Farm

The field study was conducted on a commercial dairy sheep farm breed (Lacaune and Assaf breeds). The farm is in Karditsa, Thessaly. All animals are fed a total mixed ration (TMR) (Table 7 and Table 8), and fresh drinking water is available ad libitum. The animals received 1.0 kg of chopped alfalfa hay before lambing and 1.5–2.0 kg after lambing.

### 5.3. Trial Design

**Figure 1:**
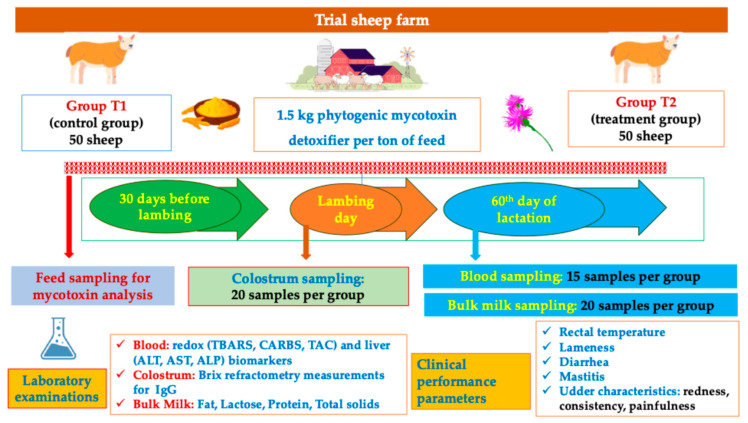
A flowchart of the experimental design.

A total of 100 dairy sheep of the Lacaune breed were included in the study. The selection criteria included the absence of pathological conditions in the last 2 months and a similar body weight. The sheep received the same total mixed ration (TMR) (see Table 1). They were randomly and equally divided into two groups: (a) T1 (control group): 50 sheep of the Lacaune breed and (b) T2 (experimental group): 50 dairy sheep of the Lacaune breed, which received an additional 1.5 kg/ton (as fed) of a multi-component mycotoxin detoxifier 30 days before lambing and during the lactation period for 60 days. During the study, the animals underwent clinical examinations once a week (Figure 1).

In this field trial, the effectiveness of an innovative mycotoxin detoxifier—BIŌNTE^®^ Quimitōx^®^ Plus (BIŌNTE Nutrition S.L., Reus-Tarragona, Spain)—was evaluated. The product comprises bentonite and sepiolite clays, phytogenic compounds (natural extracts of silymarin and curcumin), and a blend of selected yeast derivatives, including yeast cell wall and hydrolyzed yeast.

### 5.4. Sampling

During the study, feed samples weighing 500 g of the final feed were collected. The samples were analyzed for mycotoxin using lateral flow strip kits supplied by ProGnosis Biotech (Larissa, Greece).

On the 60th day of lactation, blood samples were collected from 50 sheep per group via jugular vein puncture using BD Vacutainer^®^ tubes (Becton Dickinson, Franklin Lakes, NJ, USA) containing EDTA as an anticoagulant. The samples were centrifuged at 12,000× *g* for 10 min at 4 °C to separate the plasma, which was then transferred into 1.5 mL microcentrifuge tubes and stored at −80 °C until further laboratory analysis.

Fifty pre-suckle colostrum samples (5 mL) per group were collected immediately after lambing from all ewes in each group for measurements by Brix refractometry. After cleaning the teats, colostrum was expressed 2–3 times at a 45° angle directly into sterile universal containers. Samples were placed on ice within one hour of collection, transported to the laboratory, and stored at −20 °C until further analysis.

Fifty milk samples (50 mL) were also taken per group at the 60th day of lactation in the early morning during regular milking and at least 30 min after milking. The milk samples were collected directly from the cooling tank. Before the milk samples were taken, the teats and udders of study animals were cleaned with cotton towels soaked in 70% methanol to collect the milk samples aseptically. Afterwards, they were placed in portable refrigerators at 0.0 to 4.0 °C using ice packs. All samples were sent to the laboratory within a maximum of 24 h using dry ice and freezing gels.

### 5.5. Blood and Milk Laboratory Exams for Redox and Liver Biomarkers

Plasma levels of thiobarbituric-acid-reactive substances (TBARS), protein carbonyls (CARBS), and total antioxidant capacity (TAC) were assessed using commercial assay kits (Cayman Chemical, Ann Arbor, MI, USA) in accordance with the manufacturer’s protocols. Additionally, plasma total protein, alanine aminotransferase (ALT), aspartate aminotransferase (AST), and alkaline phosphatase (ALP) concentrations were measured spectrophotometrically using commercially available kits.

### 5.6. Colostrum and Milk Laboratory Exams

Brix refractometry has been shown to correlate strongly with direct IgG measurements obtained through ELISA in sheep colostrum samples [43,48]. In this study, IgG concentrations were indirectly estimated using an analog hand-held portable Brix refractometer (serial number: 303025). Once the colostrum samples reached room temperature, they were thoroughly mixed using a vortex mixer and then analyzed. Calibration of the refractometer was performed with distilled water prior to testing, after every 50 samples, and whenever the laboratory’s ambient temperature shifted by more than 5 °C.

For measurement, 2–3 drops of colostrum or serum at room temperature (24 °C) were placed on the refractometer’s test well, and readings were recorded. All samples were tested in duplicate, and the average value was used. To prevent contamination, the refractometer well was cleaned between each sample to remove residual fat. The Brix refractometer used had a measurement range of 0–30%, and all measurements were conducted by a single trained individual. Colostrum quality was classified into three categories based on Brix values, as described by Hamer et al. [49]: ‘poor’ (<22%), ‘fair’ (22–26%), and ‘good’ (>26%).

Milk samples were analyzed for fat, protein, lactose, and total solids using a Milkoscan–Fosomatic instrument (A/S N Foss Electric, Hillerød, Denmark). Samples were kept at temperatures below 4 °C, transported to the university laboratory within one hour of collection, and analyzed immediately upon arrival.

### 5.7. Quantification of Mycotoxins in Feed

The collected feed samples were analyzed and quantified for mycotoxin at BIŌNTE Nutrition S.L. (Reus, Spain).

Samples were stored at −20 °C upon arrival to preserve their status until the analysis. Feed samples were prepared for analysis. Mycotoxins were present in feed samples using lateral flow kits based on immunochromatography. Thus, feed samples were analyzed to determine DON, FUB, AF, OTA, ZEN, and T2/HT2 levels.

Feed samples were analyzed using lateral flow strip kits supplied by ProGnosis Biotech (Larissa, Greece). Briefly, previously homogenized feed samples were ground until a fine powder was obtained. A total of 5 g of the obtained powder was weighed into a 50 mL falcon tube and 15 mL of the extraction solution (included in the kit) was added. The mixture was vortex shaken for 2 min. An aliquot (1 mL) of the supernatant was transferred to a 1.5 mL Eppendorf tube and centrifuged at 12,500 rpm for 2 min. A total of 100 µL of the supernatant was added to a dilution tube (included in the kit) and further mixed. Finally, 100 µL of the diluted extract was added to a microwell containing the lyophilized gold particles and homogenized using the micropipette until completely diluted. Once completely diluted, the strip was introduced. Then, 3 min after the introduction of the strip, it was retired from the microwell, and the strip’s cotton pad was peeled before its quantification using a 3PR-Mini instrument from ProGnosis. The quantification was performed using the S-Flow software (2.03.111) from the same company.

### 5.8. Recorded Parameters

Three observers with some previous experience of scoring clinical health parameters in sheep were selected. All evaluators received standardized training prior to the study to ensure consistency during the clinical assessment. To minimize observer bias, the assessment was conducted by the same trained staff throughout the study, and all observers were blinded to group allocation. This approach ensured reproducibility and reduced the risk of inter-observer variability. Scoring of udder health parameters, diarrhea, lameness, and temperature was performed by trained personnel unaware of whether animals belonged to the control or treatment group. The following clinical parameters were recorded during the trial period:

- *Temperature:* Rectal temperature was measured within the first 24 h after lambing by a standard digital thermometer with the following scoring system: 0 = normal (up to 40 °C); 1 = 40–41 °C, indicating slight fever; and 2 = 41.5–42 °C, indicating high fever.
- *Lameness*: Sheep were assessed in an evaluation pen, with two animals placed together at a time to minimize the effects of behavioral isolation. While pen size and shape varied across farms, each was sufficiently spacious—at least approximately 2 m^2^—to allow the animals to move freely and permit proper gait assessments. Upon entry into the pen, each sheep’s gait was evaluated, with lameness defined as a score of 2 or higher. Lameness was scored on a four-point scale according to the AWIN welfare assessment protocol for sheep based of the following scoring system: 0 = no lame, 1 = minor lameness, 2 = lame, and 3 = severe lameness [50].
- *Diarrhea*: A diarrhea scoring system was used to assess the consistency of sheep feces: a score of 1 indicated normal pellet-shaped feces; a score of 2 represented soft feces resembling a cow pat; and 3 denoted diarrhea, characterized by semi-liquid feces [51,52].
- *Mastitis*: The scoring system used for mastitis determination was moderated according to Phythian et al. [53]. Mammary glands were palpated for areas of focal or diffuse thickening, swelling, heat, pain, or discomfort (0—No mastitis or lesions present in any gland; 1—Mild mastitis and/or minor lesions/one gland affected by mastitis; 2—Mastitis and/or severe lesions/both glands affected by mastitis).
- *Udder characteristics*: In addition, mammary gland redness (0 = normal skin color, 1 = moderate redness, and 2 = pronounced redness), consistency (0 = loose, 1 = elastic, and 2 = firm), and pain sensitivity (0 = no pain, 1 = mild pain, and 2 = severe pain) were assessed. Specifically, the pain assessment was based on using behavioral indicators as these provide sensitive and non-invasive measures of pain, such as lip curling, trembling, teeth grinding, vocalizations, abnormal postures, or changes in posture, without moving or making contact to a painful body area [54,55,56].

### 5.9. Statistical Analysis

In this study samples from 100 animals were collected. Samples were equally split to two groups (T1 and T2), and a series of parameters for each animal was measured on ordinal and continuous scales, depending on the variable.

To evaluate statistically significant differences between the two groups for the variables measured on an ordinal scale, Fisher’s exact test, at a significance level of 0.05, was used in the R Studio Programming language (2025.05.1+513) [57]. The Shapiro–Wilk test was applied for the assessment of data normality. The oxidative stress biomarkers and biochemical parameters in plasma were evaluated by applying the independent samples t-test, while the Wilcoxon rank sum test for non-parametric data was used at a significance level of 0.05 for the rest of the variables measured on a continuous scale (e.g., fat, protein, etc.). The obtained results of plasma and milk samples were expressed as the mean values ± standard deviation (SD). Lastly, the examined oxidative stress biomarkers in the milk sample were compared by repeated measures ANOVA (RM-ANOVA) at each sampling point (pre-feeding, during feeding, and post-feeding) after Bonferroni’s adjustment for multiplicity.

## Figures and Tables

**Table 1:** Contingency table, summary statistics, and reported *p*-values for group comparisons of clinical study parameters.

Variable	Variable Scale	Groups				*p*-Value
		T1 (Control Group)		T2 (Experimental Group)		
		N	Mean/Median ± SD (Min–Max)	N	Mean/Median ± SD (Min–Max)	
Temperature (24 h)	0—Normal	17	0.88/1 ± 0.76 (0–2)	28	0.56/0 ± 0.74 (0–2)	0.08
	1—Slight fever	20		13		
	2—High fever	11		7		
Diarrhea score	0—normal feces	44	0.083/0 ± 0.28 (0–1)	43	0.1/0 ± 0.31 (0–1)	1
	1—“soft” feces	4		5		
Lameness	0—No lameness	35	0.33/0 ± 0.6 (0–2)	41	0.15/0 ± 0.36 (0–1)	0.18
	1—Minor lameness	10		7		
	2—Lameness	3		0		
Mastitis	0—No mastitis or lesions present	25	0.65/0 ± 0.76 (0–2)	30	0.46/0 ± 0.65 (0–2)	0.41
	1—Mild mastitis and/or minor lesions	15		14		
	2—Mastitis and/or severe lesions	8		4		
**Udder characteristics**						
Formation/Regression	0—In lactation	21	0.66/1 ± 0.66 (0–2)	36	0.25/0 ± 0.44 (0–1)	0.002 *
	1—Poorly formed/in regression	22		12		
	2—Not formed/with low milk production	5		0		
Redness	0—Physiological skin color	17	0.81/1 ± 0.7 (0–2)	29	0.4/0 ± 0.49 (0–1)	0.001 *
	1—Moderate redness	23		19		
	2—Strong redness	8		0		
Consistency	0—Loose	16	0.71/1 ± 0.54 (0–2)	32	0.35/0 ± 0.53 (0–2)	0.002 *
	1—Elastic	30		15		
	2—Solid	2		1		
Painfulness	0—Not painful	17	0.96/1 ± 0.82 (0–2)	25	0.63/0 ± 0.73 (0–2)	0.12
	1—Low grade painful	16		16		
	2—High grade Painful	15		7		

Symbol (*) indicates statistical significant difference (*p* ≤ 0.05).

**Table 2:** Contingency table, summary statistics, and reported *p*-values for group comparisons of study parameters in terms of colostrum/milk quality.

Variable	Variable Scale	Groups				*p*-Value
		T1 (Control Group)		T2 (Experimental Group)		
		N	Mean/Median ± SD (Min–Max)	N	Mean/Median ± SD (Min–Max)	
Brix Thresholds	Poor (<22%)	15	23.33/23 ± 3.51 (17–29)	3	26.5/28 ± 2.8 (19–29)	0.004 *
	Fair (22–26%)	21		13		
	Good (>26%)	12		32		
Fat		5.97/5.85 ± 0.4 (5.44–6.98)		6.63/6.6 ± 0.38 (5.68–7.38)		<0.001 *
Lactose		4.88/4.86 ± 0.1 (4.76–5.16)		4.89/4.88 ± 0.15 (4.58–5.09)		0.53
Protein		5.48/5.47 ± 0.15 (5.27–5.88)		5.56/5.47 ± 0.26 (5.26–6.24)		0.9
Total solids		17.1/16.96 ± 0.32 (16.72–17.78)		17.56/17.48 ± 0.46 (16.81–19.06)		<0.001 *

Symbol (*) indicates statistical significant difference (*p* ≤ 0.05).

**Table 3:** Mycotoxin analysis in feed during the trial.

Mycotoxins	Concentration (μg/kg)	Maximum Level (μg/kg) According to EU Regulation *	Technical Limit (μg/kg)
AF-B1	26.66	20	5
FUM (B1 + B2)	>4000	20,000–50,000	1500
OTA	1.62	250	50
DON	<150	2000	300
ZEN	<35	500	100
T2/HT-2	<40	250	50

* European Commission (2006, 2011, 2013, and 2019) [17,18,19,20].

**Table 4:** Evaluation of oxidative stress biomarkers in plasma.

Parameters	T1 (Control Group)	T2 (Experimental Group)	*p* Value
TBARS (μM MDA)	3.84 ± 0.31 ^a^	2.17 ± 0.26 ^b^	<0.001
Carbs (nmol/mg protein)	1.85 ± 0.13 ^a^	1.25 ± 0.19 ^b^	<0.014
TAC (mM Trolox Equivalent)	0.79 ± 0.01 ^a^	0.87 ± 0.03 ^a^	0.211

Different superscripts display the presence of statistical significance (*p* < 0.05).

**Table 5:** Assessment of plasma biochemical parameters.

Parameter	T1 (Control Group)	T2 (Experimental Group)	*p* Value
Alanine aminotransferase (ALT, U/L)	38.14 ± 0.78 ^a^	17.73 ± 0.63 ^b^	0.011
Aspartate aminotransferase (AST, U/L)	110.31 ± 1.33 ^a^	79.51 ± 1.16 ^b^	0.027
Alkaline phosphatase (ALP, U/L)	136.98 ± 1.42 ^a^	97.89 ± 1.69 ^b^	0.021

Different superscripts in the same line indicate the presence of statistical significance (*p* < 0.05).

**Table 6:** Assessment of oxidative stress biomarkers in milk.

Parameters	T1 (Control Group)	T2 (Experimental Group)	*p* Value
TBARS (μM MDA)	21.03 ± 1.32 ^a^	9.88 ± 0.89 ^b^	<0.001
CARBS (nmol/mg protein)	0.48 ± 0.11 ^a^	0.24 ± 0.04 ^b^	<0.001

Different superscripts in the same line indicate the presence of statistical significance (*p* < 0.05).

**Table 7:** Ingredients and chemical composition of study animals’ diets.

Ingredients	kg per Ton
Alfalfa hay	300
Corn silage	200
Soybean meal	100
Corn grain	150
Barley grain	100
Sunflower meal	60
Wheat brans	72
Fat	5
Molasses	15
Mineral and vitamin premix *	10
Monofos	7
Sodium bicarbonate	3
Salt	4
Adhesive	3
Calcium carbonate	21
**Chemical composition**	
Energy (KJ/100 g)	1525
Crude protein (%)	17.7
Crude fiber (%)	3.32
Crude fat (%)	3.38
Ash (%)	6.36
Sodium (Na) (%)	0.33
Calcium (Ca) (%)	1.19
Phosphorus (P) (%)	0.55
Magnesium (Mg) (%)	0.28

* Analysis of mineral and vitamin premix per Kgr.

**Table 8:** Analysis of mineral and vitamin premix per Kgr.

Vitamins		Trace Elements	
Vitamin A (3a672a)	15,000 I.U.	Sodium selenite (Se)	0.35 mg
Vitamin D_3_	2500 I.U.	Iron (II) sulfate monohydrate	120 mg
Vitamin Ε (dl-a tocopheryl acetate)	50 mg	Manganese (II) oxide	105.4 mg
		Zinc oxide	78 mg
Vitamin Β_1_	5 mg	Co (II) carbonate	0.3 mg
Vitamin Β_2_	0.8 mg	Iodine (calcium Iodate)	4 mg
Vitamin B_6_	0.35 mg	Zinc chelate of glycine hydrate	34 mg
Vitamin B_12_	20 mcg	Selenium (selenomethionine)	0.07 mg
Niacin	24.75 mg	Antioxidants: (Ε310)	0.4 mg
Biotin	2.4 mg	ΒHΤ (Ε321)	0.2 mg
*Saccharomyces cerevisiae*	750 × 10^7^ cfu	1a330 Citric acid	0.2 mg

## Data Availability

The original contributions presented in this study are included in the article/Supplementary Material. Further inquiries can be directed to the corresponding author.

## Supplementary Materials

The following supporting information can be downloaded at https://www.mdpi.com/article/10.3390/toxins17080425/s1. Table S1. Assessment of clinical signs, lameness, mastitis, and milk composition in ewes by treatment group.
